# Albinism in the largest extant amphibian: A metabolic, endocrine, or immune problem?

**DOI:** 10.3389/fendo.2022.1053732

**Published:** 2022-11-28

**Authors:** Liming Chang, Wei Zhu, Jianping Jiang

**Affiliations:** CAS Key Laboratory of Mountain Ecological Restoration and Bioresource Utilization & Ecological Restoration and Biodiversity Conservation Key Laboratory of Sichuan Province, Chengdu Institute of Biology, Chinese Academy of Sciences (CAS), Chengdu, China

**Keywords:** albinism, autoimmune, disease model, genetic polymorphism, melanin, transcriptomics

## Abstract

**Background:**

Pigment regression is an intriguing phenomenon that can be caused by disorders in melanin metabolism or endocrine regulation, or by autoimmune disorders. Albino animals serve as excellent models for the study of the genetic determination of morphology, particularly the evolution of and molecular mechanisms underlying chromatophore-related diseases in animals and humans.

**Material and Methods:**

The artificial culture of *Andrias davidianus*, the largest extant amphibian, is flourishing in China due to the great ecological and economic value of this animal. Approximately 0.1% of individuals express an albino phenotype accompanied by delayed somatic growth and mortality at early developmental stages. In this study, brain and skin transcriptomics were conducted to study the underlying molecular basis of the phenotype.

**Results:**

The results indicated decreased transcription of genes of melanin synthesis. Interestingly, MHC I isotypes and immune-related pathways accounted for the primary transcriptional differences between groups, suggesting that the albino phenotype represents a systematic immune problem to a far greater extent than a pigmentation defect. Albino individuals exhibited shifted transcription of MHC I isotypes, and the albino-specific isotype was characterized by increased charges and decreased space in the antigen- binding pocket, implying a drastic change in antigen specificity and a potential risk of autoimmune disorders.

**Conclusion:**

These results suggest an association between the albino phenotype and MHC I variants in *A. davidianus*, which could serve as a convenient model for vitiligo or other autoimmune diseases.

## Introduction

Pigment regression due to genetic factors is commonly observed in vertebrates (1, 2). This can appear as a morphological trait of an entire population or species adapted to specific environments (e.g., darkness in caves or soil layers) (3, 4). More often, hypopigmentation is a minority feature appearing in members of a population or species carrying allele variants (5, 6). Typical examples are the pigmentary disorders occurring in humans, such as vitiligo, piebaldism, and albinism. This raises the question as to which internal factors regulate the pigment cells and pigmentation processes and thus govern hypopigmentation phenomena in vertebrates.

From a mechanistic perspective, hypopigmentation is always associated with mutations in genes that either participate in melanin synthesis (e.g., tyrosinase and melanosomal transmembrane protein) (4, 5) or are responsible for signal regulation in melanin synthesis and melanophore proliferation (e.g., melanocortin receptors and agouti) (3, 7). In the latter type of depigmented morphs, hypopigmentation is accompanied by additional physiological outcomes due to the potential crosstalk between regulation pathways (8, 9). Pigmentation is linked to the levels of many endocrine factors and the activation of related signal transduction pathways, including the melanocyte-stimulating hormones (MSHs), adrenocorticotropic hormone, steroid hormones, and prostaglandins (10). Among these factors, alpha-MSH is the best described; its precursor, proopiomelanocortin, is synthesized in the brain and pituitary primordium (11). Additionally, hypopigmentation can alternatively arise as a result of genetic variation in cellular processes indirectly related to melanin systems. One typical example of this type of case is vitiligo (12), an autoimmune skin disease characterized by patches of depigmentation caused by the destruction of melanophores (13). This disease is linked to genetic polymorphism of the MHC regions (14–17).

**Figure 1:**
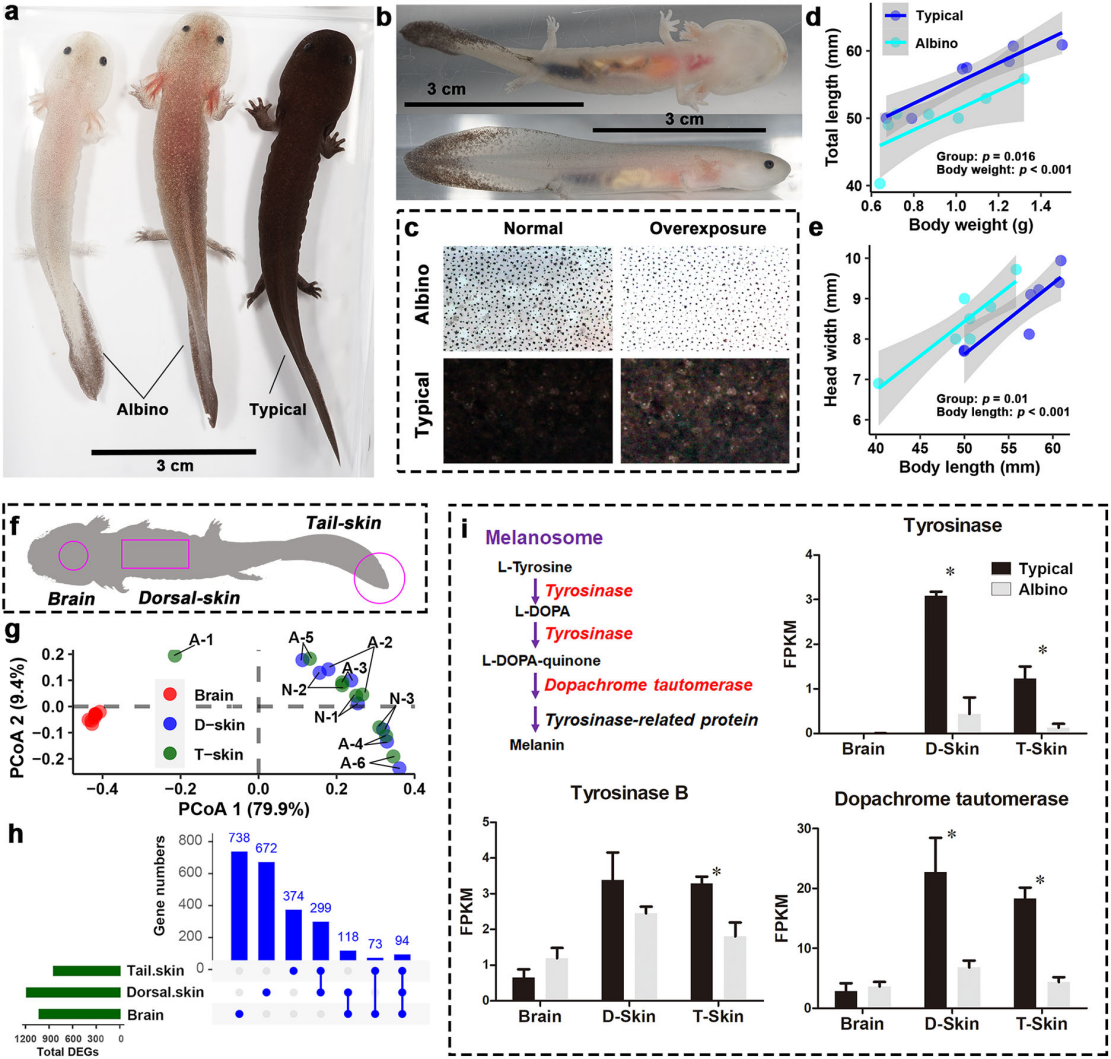
Morphological and transcriptional differences between A'lbino and typical *A davidianus.* **(A, B)** Appearance of albino individuals. **(C)** Comparison of dorsal skin pigment. **(D, E)** Differences between groups in overall length **(D)** and head width **(E)**. **(F)** Transcriptome sampling scheme. **(G)** PCoA scatter plot presenting the similarity in transcriptional profiles between samples. Dorsal and tail skin tissues with the same labels are samples from the same individuals. ‘A’ and ‘B’ in the labels denote ‘Albino’ and ‘typical’, respectively. Note that dorsal and tail skins from the same individuals are always associated with more similar transcriptomes, indicating a high degree of technical stability in our RNA-seq data. **(H)** Statistics on DEGs in different tissues. **(I)** Transcriptional variations of genes in melanin synthesis. Asterisks denote significant differences between groups (at a threshold of *p* < 0.05, Student’s *t* test).

Depigmented animals are excellent models for investigation of the genetic determination of the corresponding morphological traits, and studies of such animals may provide evolutionary and mechanistic insights into animal and human chromatophore-related diseases (e.g., vitiligo, albinism, and melanoma). Although recent investigations have deepened our understanding of these diseases, many questions surrounding their initiation and progression remain to be answered (13), and proper animal models are important as a means of studying their pathogenesis and facilitating the discovery and evaluation of therapeutic interventions (18). The Chinese giant salamander (Cryptobranchidae: *Andrias davidianus*) is the largest extant amphibian species (19). Its ancestors diverged from other amphibians over 170 million years ago during the Jurassic Period (20), making it one of the oldest families on the amphibian tree of life. The artificial culture of *A. davidianus* is flourishing in China due to the great ecological and economic value of this animal. In practice, approximately 0.1% of individuals of the species express an albino phenotype of the entire body (**Figure 1A**), characterized by semitransparent skin with reduced skin pigmentation (**Figures 1B, C**). These albino individuals always suffer additionally from other physiological problems. For example, they grow more slowly than their typical siblings and do not live through their first year. The genetic basis of their albino phenotype has potential significance in the aquaculture breeding of *A. davidianus* and in the development of disease models. In this study, the gene transcriptional profiles of the dorsal and tail skin and brain are compared between albino and typical individuals to provide insight into the molecular basis of depigmentation and associated physiological abnormalities.

## Methods and materials

### Sample collection

Artificially-bred *A. davidianus* were collected from a farm located in Hongya, Sichuan Province, China (103°10′05′′E, 29°52′36′′N). Fertilized eggs of this species take approximately 70 days to develop to the stage at which formation of the fifth digit in the hindlimb occurs (stage 46 according to the chronological table suggested by Shi and Boucaut (21)) (22). These larvae take more than 300 days to complete their metamorphosis (characterized by disappearance of the gills) after hatching at 15 °C. In this study, albino and typical individuals (n = 7 per group) were collected approximately 70–80 days after hatching. All the larvae were at stage 46 (21) when they were collected. The albino phenotype appeared sporadically across clutches, and thus the seven albino individuals all had different parents. Correspondingly, the typical individuals were randomly collected from different clutches. Their body weight, body length, head width, tail length, head length, orbital distance, nasal distance, and eye diameters were measured. After having been euthanized with MS-222, the larvae were dissected to collect the dorsal skin, the tail skin, and the brain. All samples were stored at -80 °C.

### Transcriptome analysis

Transcriptome measurements were obtained from the brain and the dorsal and tail skin of six albino and three typical individuals. The brain transcriptome was included as the brain is a central organ in the regulation of pigmentation in amphibians. Previously-described protocols were followed for RNA extraction, purification, cDNA library construction, filtration, assembly, annotation, and gene expression quantification (23). RNA-seq was performed on an Illumina HiSeq 4000 platform by Annoroad (Beijing); paired-end reads were generated. A previously-reported multi-organ whole-length transcriptome was used as the reference genome for transcript identification, annotation, and gene expression quantification (24), and FPKM values were calculated for each unigene (see gene expression matrix in **Table S1**). This approach may improve the accuracy and reliability of the transcriptional quantification in comparison to a *de novo* assembly approach. Differently expressed genes (DEGs) were identified by Student’s *t* test, and functional enrichment analyses were conducted by querying DEGs against the KEGG database (based on KOBAS 3.0, with default parameters) (25).

### Sequence comparison and phylogenetic analyses

Sequences of targeted genes were retrieved from Genbank or from our transcriptome database. Sequence alignment was performed using Clustal X2, and further edits were made using GeneDoc. A maximum likelihood tree was constructed using MEGA7 with default parameters.

### Prediction of 3D protein models

3D models of MHC class I proteins were predicted on the SWISS-MODEL server (https://www.swissmodel.expasy.org/) with “marsupial MHC I (7edo.2.A)” as the model. Analysis of 3D models was performed using Swiss- Pdb Viewer.

### Statistical analysis

Basic statistical analyses were conducted using IBM SPSS 22.0 (SPSS Inc., Chicago, USA). Kolmogorov–Smirnov tests were conducted to assess the deviation of the data from normal distribution. Inter-group differences in body length and width were analyzed *via* ANCOVA with body weight as a covariate. Inter-group differences in other body traits (using relative values) were analyzed *via* Student’s *t* test. Dissimilarity in transcriptomes was operationalized using the Bray–Curtis distance, which was calculated using the vegdist function of the R package Vegan. Subsequently, PERMANOVA (using the Adonis function of the Vegan package) was employed to identify differences in transcriptomes between the groups, and PCoA was conducted to present the differences between groups. Graphs were generated using GraphPad Prism 5 or the R package ggplot2 (26).

## Results

The albino individuals had comparable body width to typical individuals with similar body weight, but their bodies were smaller in length, resulting in a stockier overall body shape (**Figures 1D, E**, **S1**). Their relative tail length, head length, orbital distance, and eye diameters were comparable to those of typical individuals; however, their nasal distance was much smaller (**Figure S1**). Transcriptomics was conducted for the dorsal and tail skin samples, as well as the brain (**Figure 1F**). As expected, the two types of skin tissue shared similar transcriptional profiles, which were quite distinct from that of the brain (**Figure 1G**). PERMANOVA results suggested that there was a significant inter-group difference in brain transcriptome, while the transcriptional differences between groups in the skin samples were less significant (**Figure S2**). In total, 1,023, 1,183, and 840 DEGs between the groups were identified in the brain, dorsal skin, and tail skin, respectively, among which 299 DEGs were shared by the two skin tissue types (**Figure 1H**).

**Figure 2:**
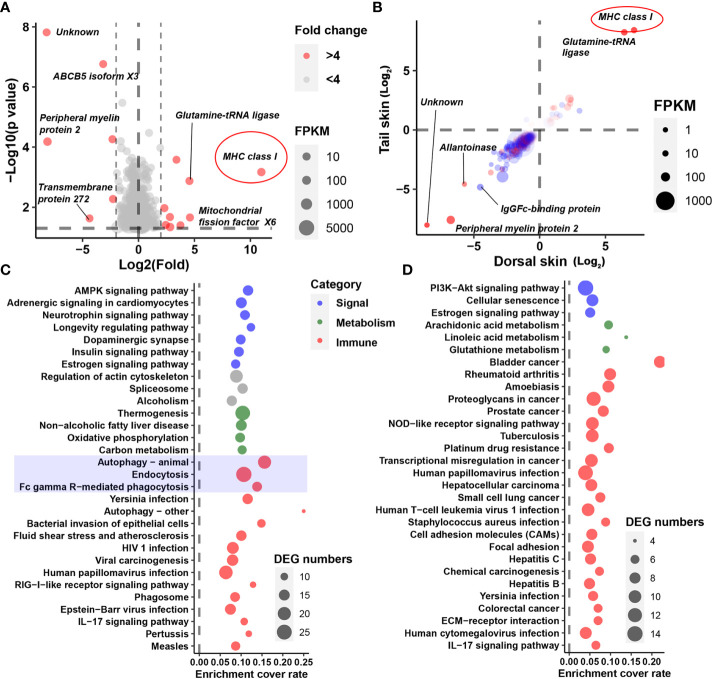
Identification of crucial DEGs and cellular processes. **(A)** The DEGs representing the greatest fold change in the brain. **(B)** Fold changes in DEGs (*p* < 0.05) in the dorsal (horizontal axis) and tail (vertical axis) skins. Red dots denote DEGs shared by all three tissue types; blue dots denote DEGs shared by the two skin tissue types. Note that most DEGs exhibit consistent trends in variation (i.e., an increase or decrease) between the two types of skin tissue, indicating the reliability of the DEGs. **(C, D)** Functional analysis based on DEGs in the brain **(C)** or those shared by the skin samples **(D)**. Only the top 30 significant items are displayed (*q* < 0.01).

Three genes involved in melanin synthesis (namely, tyrosinase, tyrosinase B, and dopachrome tautomerase) were identified among the DEGs; these exhibited decreased transcription in the skin of albino individuals (**Figure 1I**). Interestingly, however, melanin synthesis did not account for the most prominent transcriptional changes in the albino larvae. Instead, an MHC class I gene was highlighted as exhibiting the highest degree of fold changes (in the albino *vs.* the typical group) in all three types of tissue (fold changes = 2,000, 144, and 340 in the brain, dorsal skin, and tail skin, respectively; **Figures 2A, B**). Correspondingly, KEGG enrichment analysis based on brain DEGs (at a threshold of *p* < 0.05) highlighted changes in immune-related processes (e.g., autophagy, endocytosis, and Fc gamma R-mediated phagocytosis), signal transduction (e.g., the AMPK signaling pathway and adrenergic signaling in cardiomyocytes), and metabolic pathways (e.g., thermogenesis and oxidative phosphorylation) (**Figure 2C**). The skin DEGs (meeting the threshold of *p* < 0.05 in both tissue types) highlighted changes in the autoimmune and cancer pathways, e.g., rheumatoid arthritis and bladder cancer (**Figure 2D**). Furthermore, several signaling pathways (e.g., adrenergic signaling in cardiomyocytes and the estrogen signaling pathway) were also enriched by DEGs, implying the potential presence of endocrine disorders in the albino larvae.

**Figure 3:**
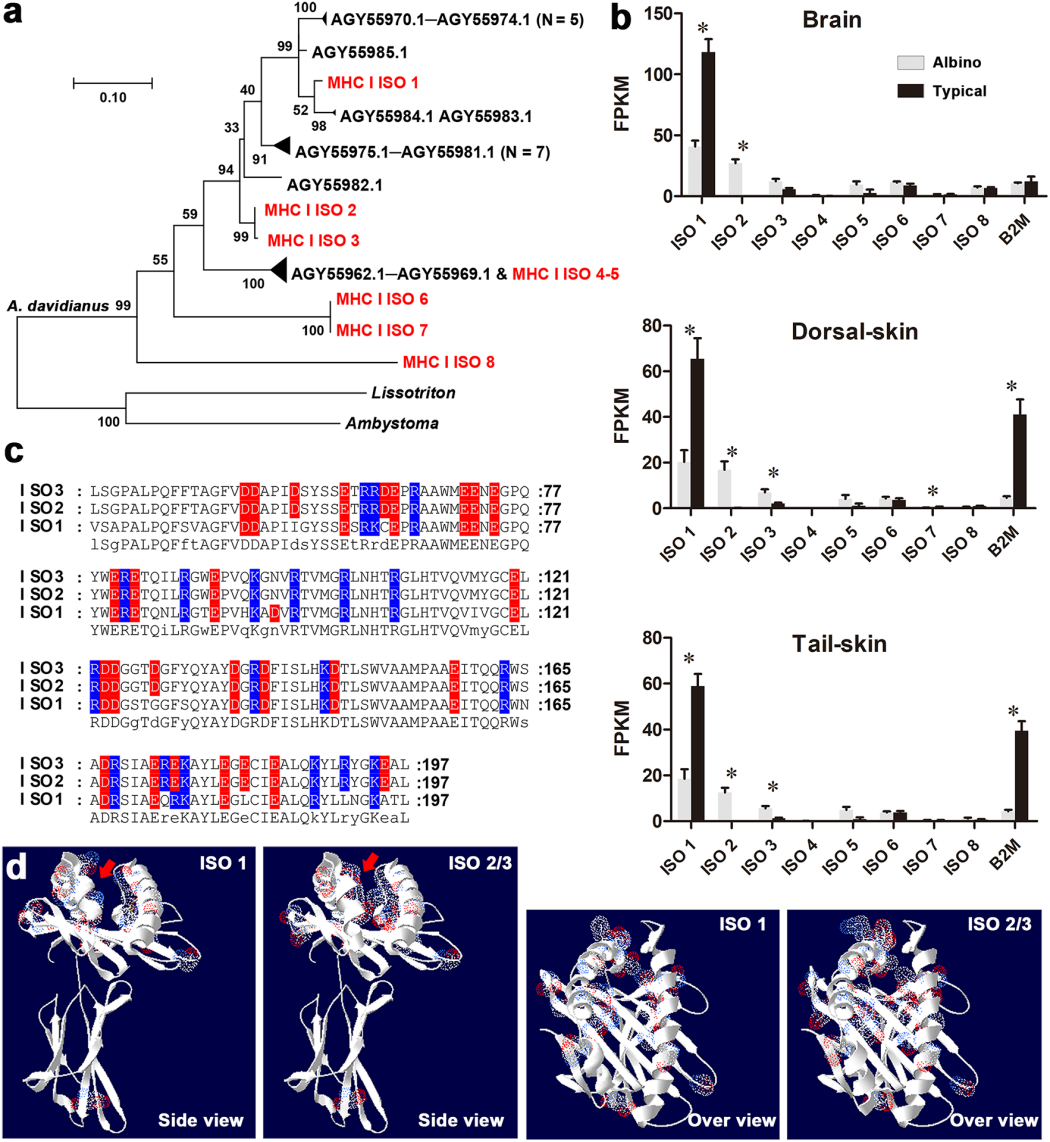
Sequence and expression analysis of MHC class I genes. **(A)** Maximum likelihood tree for MHC class I genes (see the sequences in **Table S2**). Red items denote MHC class I isotypes identified in this study; black items denote the known MHC class I genes of *A davidianus* and closely related species. **(B)** Transcriptional levels of MHC class I isotypes and B2M. Asterisks denote significant differences between groups (at a threshold of *p* < 0.05, Student’s *t* test). **(C)** Amino acid substitutions in the antigen- binding regions of MHC class I isotypes. Red and blue characters represent negative and positive charges. **(D)** 3D structure of MHC class I isotypes. The sites of amino acid substitution are highlighted by electron clouds (blue: positive residues; red: negative residues). Red arrows denote antigen- binding pockets. Note the reduced space and increased charges in the antigen- binding pockets of isotypes 2 and 3.

A total of eight MHC class I isotypes (labeled ISO 1–8) were identified in the whole-length transcriptome of *A. davidianus* (**Figures 3A**, **S3**). Isotypes ISO 1–3 and ISO 4–5 belong to different subclasses, whose members have been reported previously, while isotypes ISO 6–8 belong to new subclasses. ISO 1 is the dominating isotype expressed in the tissues of typical individuals. In albino individuals, the transcription of this isotype and β_2_ microglobulin (B2M), components of MHC I, exhibited dramatic downregulation. Meanwhile, they also exhibited notably increased transcription of ISO 2 and ISO 3 (**Figure 3B**). The sequence variances between ISO 1 and ISO 2–3 were mainly distributed in the α1 and α2 functional domains, which are responsible for antigen binding (**Figures 3C, D**). Within these domains, six neutral amino acid residues were replaced by charged ones in ISO 2/3. These substitutions increased the charges and reduced the amount of space in the antigen-binding pocket.

## Discussion

Our results suggest that the albino phenotype is associated with shorter body length and reduced relative nasal distance in *A. davidianus*. As the relative tail and head lengths are unchanged, the shorter body length can most likely be attributed to a systematic problem with growth. The biological significance of nasal distance has not been fully understood in animals, but it is a common morphological trait used in amphibian taxonomy, as it reflects the anatomic feature of the skull bone. These morphological changes may be explained by skeletal dysplasias, a class of disorders characterized by orthopedic complications and varying degrees of dwarfism or short stature (27). The morphological abnormalities observed in the albino phenotype in *A. davidianus* are not restricted to pigmentation problems. Similar morphological abnormalities have not been reported in white and albino *Ambystoma mexicanum*, which can grow and breed normally (28). The white phenotype of *A. mexicanum* is linked to genetic mutations preventing the differentiation and migration of all types of chromatophores, while the albino phenotype is due to the dysfunction of enzymes responsible for melanin synthesis (29, 30). Albino *A. davidianus* larvae do retain some melanophore and melanin throughout the entire body, particularly in the tail, whereas white and albino *A. mexicanum* are almost devoid of melanin. This suggests that different mechanisms should be expected to underlie the depigmentation phenomenon in *A. davidianus* and *A. mexicanum*.

The decreased transcription of tyrosinases and dopachrome tautomerase observed in the skin could explain the reduced pigmentation in albino *A. davidianus* (**Figure 1**). Interestingly, the transcriptional change in the brain was even more prominent than that occurring in the skin samples (**Figure S2**), although the latter contains the largest amount of melanin. In both the brain and the skin tissues, the transcription of an MHC class I gene accounted for the largest fold changes in albino individuals (**Figures 2A, B**). MHC class I molecules occur on all nucleated cells and present intracellular peptides to killer T cells in order to mediate cellular immunity, including the cytotoxic effect, phagocytosis, and inflammation. MHC class I gene variants are highly polymorphic, and their profiles and expression levels play an important role in autoimmune disorders, infectious diseases, and immunosurveillance (31, 32). In albino *A. davidianus*, the changes in the transcriptional profiles of MHC class I isotypes are consistent with the enrichment of DEGs in autophagy, phagocytosis, autoimmune, and cancer pathways in the brain and skin (**Figures 2C, D**). Functional enrichment based on DEGs also suggests the potential presence of endocrine disorders in albino individuals. However, this may be a result of autoimmune problems, despite the fact that we cannot exclude the potential role of the endocrine system in causing pigment regression, growth retardation, and increased mortality in albino *A. davidianus*. Overall, our results suggest that the albino phenotype encompasses far more than a pigmentation defect; rather, it represents a systematic problem associated with the immune system. This could explain the concomitant physiological defects, such as malformation, delayed growth, and mortality at early development (33), as immune activity can be linked to growth and development performance through both resource allocation (34) and crosstalk between regulation pathways (35).

The association between MHC class I and depigmentation has been well established in vitiligo, which is visible in the form of white spots and affects ∼1% of the world’s human population. Mounting strands of evidence support the theory that vitiligo is linked to genetic changes in MHC gene regions (14–17). High-risk MHC class I alleles can present many autoantigens, including some melanocyte proteins, and thus induce autoimmune responses against melanocytes (36). In addition to the change in antigen specificity produced by variations in coding regions, the altered expression of MHC class I and II genes, caused by genetic variation in adjacent transcriptional regulatory regions, plays an even more important role in the activation of autoimmune responses (37–39). Albino *A. davidianus* is characterized by an inverse transcriptional change to MHC I isotypes 1 and 2/3. The albino-specific MHC I isotypes 2 and 3 exhibit increased charges and decreased space in their antigen- binding pockets, implying a drastic change in their antigen specificity. Accordingly, this transcriptional shift in MHC I isotypes may explain the depigmentation occurring in albino individuals. This suggests that the albino *A. davidianus*, which has uncovered skin and is readily accessible due to large-scale farming, constitutes a potential animal model for study of the pathogenesis of and therapeutic interventions for vitiligo or other autoimmune diseases.

On this basis, further investigations may focus on the molecular mechanisms underlying the associations between MHC I isotypes and morphological and physiological defects: for example, examining how the expression of MHC I isotypes 2 and 3 affects the autoimmune response and apoptosis in different tissue and cell types. Moreover, it is also important to study the genetic structure, polymorphism, and diversity of the MHC regions of *A. davidianus* at the population level, and to identify the regulatory regions that determine the expression profile of different MHC I isotypes.

## Conclusions

Approximately 0.1% of *A. davidianus* individuals express an albino phenotype accompanied by delayed somatic growth and mortality at early developmental stages. Transcriptional analysis indicated decreased transcription of genes involved in melanin synthesis in the skin in such individuals; however, MHC I genes accounted for the most notable transcriptional changes in the brain and skin. In combination with the enrichment of autophagy, phagocytosis, autoimmune, and cancer pathways by DEGs, our results suggest that the albino phenotype represents a systematic immune problem rather than disorders in melanin synthesis or endocrine regulation of pigmentation. Moreover, albino individuals exhibited shifted transcription of MHC I isotypes, and the albino-specific isotype was characterized by increased charges and decreased space in the antigen- binding pocket, implying a drastic change in antigen specificity and the potential risk of autoimmune disorders. Overall, these results suggest an association between the albino phenotype and MHC I variants in *A. davidianus*, which lends it potential as an animal model for vitiligo or other autoimmune diseases.

## Data availability statement

The datasets presented in this study can be found in online repositories. The names of the repository/repositories and accession number(s) can be found in the article/**Supplementary Material**.

## Ethics statement

The animal study was reviewed and approved by Animal Ethical and Welfare Committee of Chengdu Institute of Biology, Chinese Academy of Sciences (permit: CIB20160305).

## Author contributions

LC and WZ, conceptualization, formal analysis, investigation, methodology, visualization, writing (original draft), manuscript review and editing. JJ, conceptualization, methodology, funding acquisition, manuscript review and editing. All authors gave their final approval for publication and agree to be held accountable for the work reported therein.

## Funding

This work was funded by the Strategic Priority Research Program of the Chinese Academy of Sciences (XDA19050201), Youth Innovation Fund Program of Chengdu Institute of Biology (CAS) (E2B1040001), Construction of Basic Conditions Platform of Sichuan Science and Technology Department (2019JDPT0020), and China Biodiversity Observation Networks (Sino BON–Amphibian and Reptile).

## Acknowledgments

We thank Sheng-Chao Shi for assistance in obtaining photographs and Chun-Lin Zhao for collecting animals.

## Conflict of interest

The authors declare that the research was conducted in the absence of any commercial or financial relationships that could be construed as a potential conflict of interest.

## Publisher’s note

All claims expressed in this article are solely those of the authors and do not necessarily represent those of their affiliated organizations, or those of the publisher, the editors and the reviewers. Any product that may be evaluated in this article, or claim that may be made by its manufacturer, is not guaranteed or endorsed by the publisher.
