# Supplementary material for: Albinism in the largest extant amphibian: A metabolic, endocrine, or immune problem?
Source: Front Endocrinol (Lausanne). 2022 Nov 28;13:1053732. doi: 10.3389/fendo.2022.1053732 (PMC9742363; doi:10.3389/fendo.2022.1053732)
Supplement: Supplementary file 1 [file DataSheet_1.pdf]

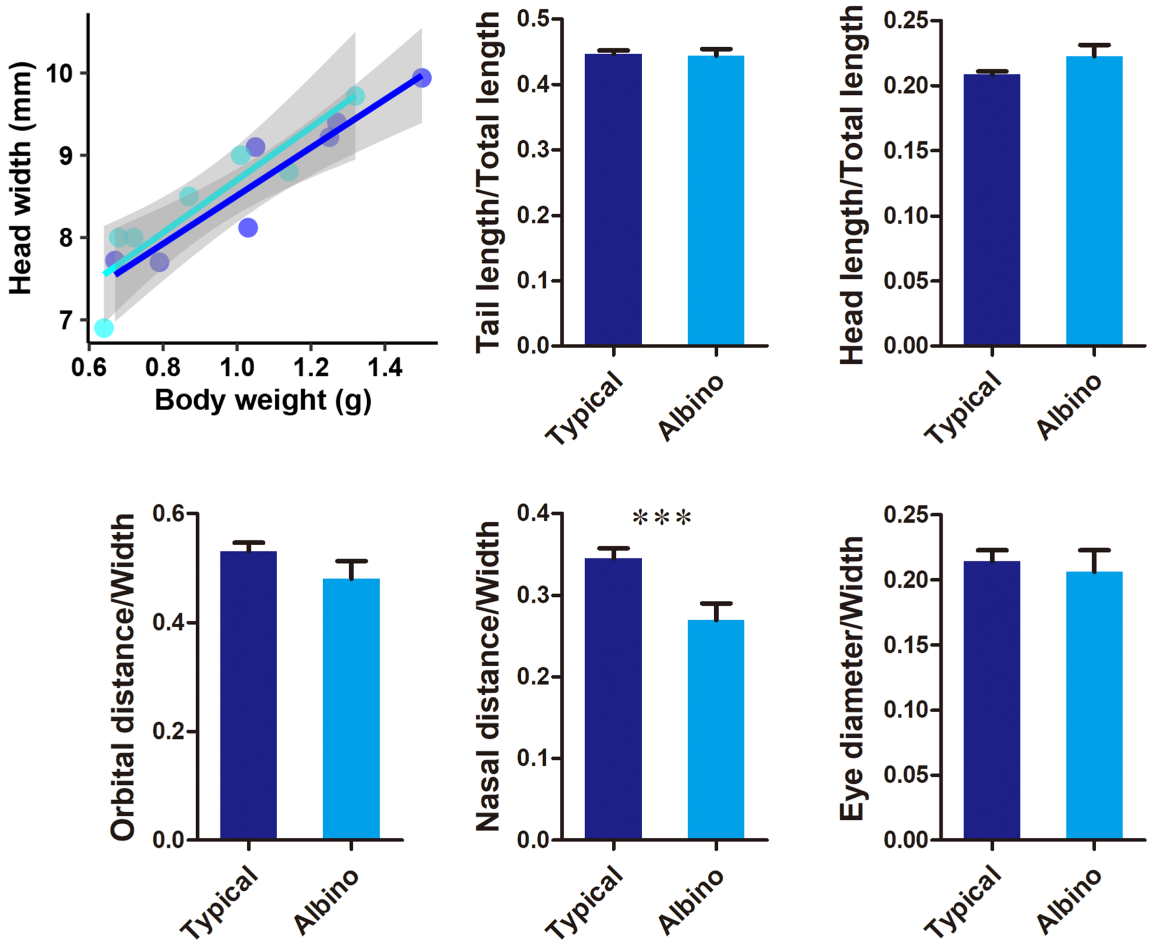

Figure S1 Comparison of body traits between normal and albino individuals. ***, $p < 0.001$, Student's t test. Blue and cyan colors denote typical and albino individuals, respectively.

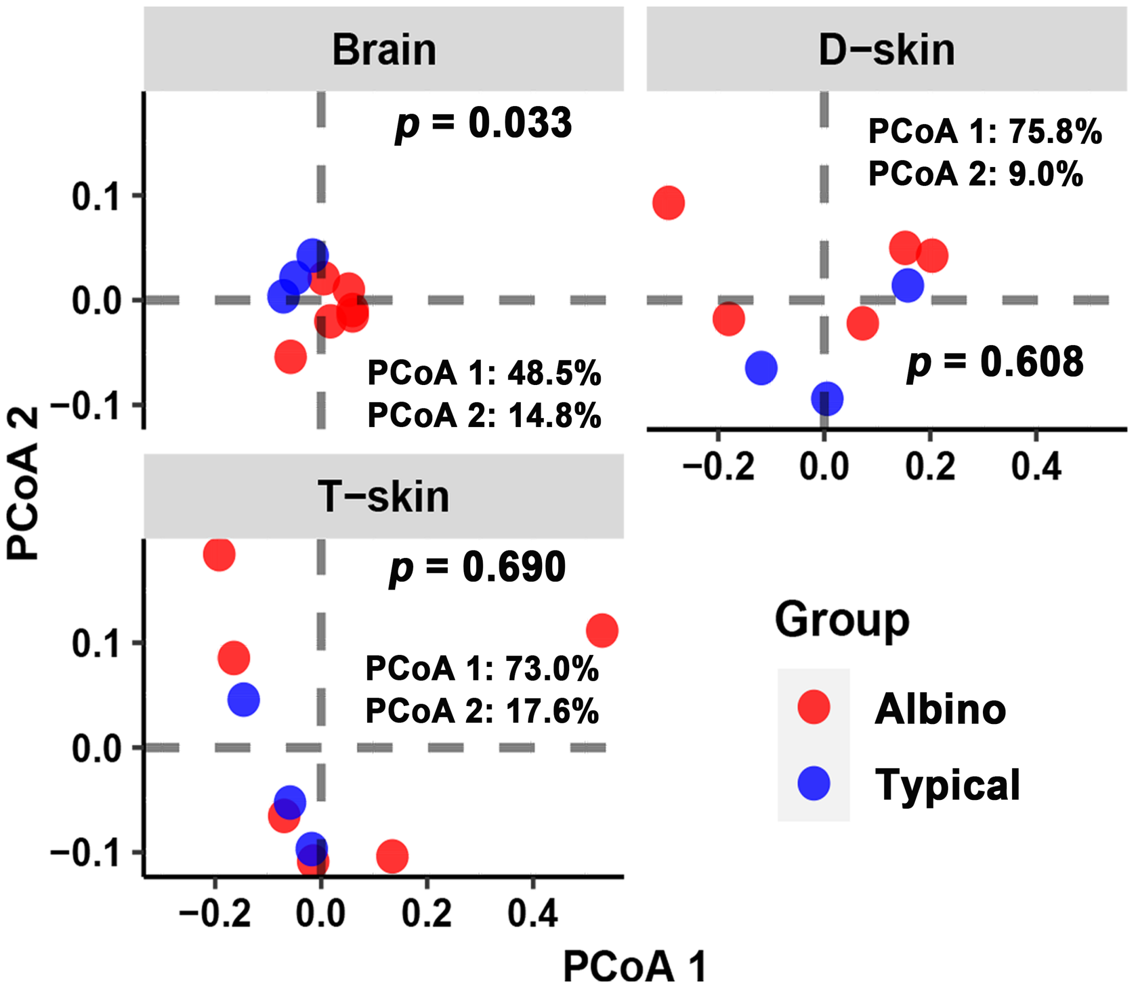

Figure S2 Comparison of tissue transcriptional profiles between normal and albino individuals. The differences between groups were examined by PERMANOVA.

```
                 *        20         *        40
ISO3 : MMSRTTLMPLLLVAVCLRGACAGSHSLRYFYSALSGPALPQFFT :  44
ISO2 : MMSRTTLMPLLLVAVCLRGACAGSHSLRYFYSALSGPALPQFFT :  44
ISO1 : MMCRTTLMPLLLVAVCLRGACAGSHSLRYFYSAVSAPALPQFSV :  44
       MMsRTTLMPLLLVAVCLRGACAGSHSLRYFYSA6SgPALPQFft

             *        60         *        80
ISO3 : AGFVDDAPIDSYSSETRRDEPRAAWMEENEGPQYWERETQILRG :  88
ISO2 : AGFVDDAPIDSYSSETRRDEPRAAWMEENEGPQYWERETQILRG :  88
ISO1 : AGFVDDAPIIGYSSESRKCEPRAAWMEENEGPQYWERETQNLRG :  88
       AGFVDDAPIdsYSSE3R4dEPRAAWMEENEGPQYWERETQiLRG

         *        100        *        120       *
ISO3 : WEPVQKGNVRTVMGRLNHTRGLHTVQVMYGCELRDDGGTDGFYQ : 132
ISO2 : WEPVQKGNVRTVMGRLNHTRGLHTVQVMYGCELRDDGGTDGFYQ : 132
ISO1 : TEPVHKADVRTVMGRLNHTRGLHTVQVIVGCELRDDGSTGGFSQ : 132
       wEPVqKg1VRTVMGRLNHTRGLHTVQV6yGCELRDDGgTdGFyQ

            140        *        160        *
ISO3 : YAYDGRDFISLHKDTLSWVAAMPAAEITQQRWSADRSIAEREKA : 176
ISO2 : YAYDGRDFISLHKDTLSWVAAMPAAEITQQRWSADRSIAEREKA : 176
ISO1 : YAYDGRDFISLHKDTLSWVAAMPAAEITQQRWNADRSIAEQRKA : 176
       YAYDGRDFISLHKDTLSWVAAMPAAEITQQRWsADRSIAEreKA

        180        *        200        *        220
ISO3 : YLEGECIEALQKYLRYGKEALQRRERPETMVSHHKSGASNARLT : 220
ISO2 : YLEGECIEALQKYLRYGKEALQRRERPETMVSHHKSGASNARLT : 220
ISO1 : YLEGLCIEALQRYLLNGKATLQRRERPETMVSHHKSGASNARLT : 220
       YLEGeCIEALQ4YLryGKeaLQRRERPETMVSHHKSGASNARLT

                 *        240        *        260
ISO3 : CHAYKFYPREIEVKWFRNGVEMPAKFLPQTLPNPDGTYQIKTTV : 264
ISO2 : CHAYKFYPREIEVKWFRNGVEMPAKFLPQTLPNPDGTYQIKTTV : 264
ISO1 : CHAYKFYPREIEVKWFRNGVEMPAKFLPQTLPNPDGTYQIKTTV : 264
       CHAYKFYPREIEVKWFRNGVEMPAKFLPQTLPNPDGTYQIKTTV

             *        280        *        300
ISO3 : DIPEGEEEMYICRVDHSSLDNALDVKYEKKALPIGLIIGAIAGV : 308
ISO2 : DIPEGEEEMYICRVDHSSLDNALDVKYEKKALPIGLIIGAIAGV : 308
ISO1 : EVPEGEEEMYICRVDHSSLDNALDVKYEKKALPIGLIIGAIAGV : 308
       d6PEGEEEMYICRVDHSSLDNALDVKYEKKALPIGLIIGAIAGV

         *        320        *        340       *
ISO3 : VVLLAAAIGGIVIWRKR-SGQGGYAAANK-----DAGI----- : 340
ISO2 : VVLLAAAIGGIVIWRKRSAGQGGYAAANKGPINADSSSSTASA : 351
ISO1 : VVLLAAAIGGIVIWRKRSAGRGGYAAANKGPINGDSSSSTASA : 351
       VVLLAAAIGGIVIWRKRsaGqGGYAAANKgpin Dsssstasa
```

Figure S3 Sequence alignment of MCH I isoforms of *A. davidianus*.
